# Supplementary figures and images for: Identification and Comparative Analysis of Long Non-Coding RNA in the Skeletal Muscle of Two Dezhou Donkey Strains
Source: Genes (Basel). 2020 May 4;11(5):508. doi: 10.3390/genes11050508 (PMC7288655; doi:10.3390/genes11050508)

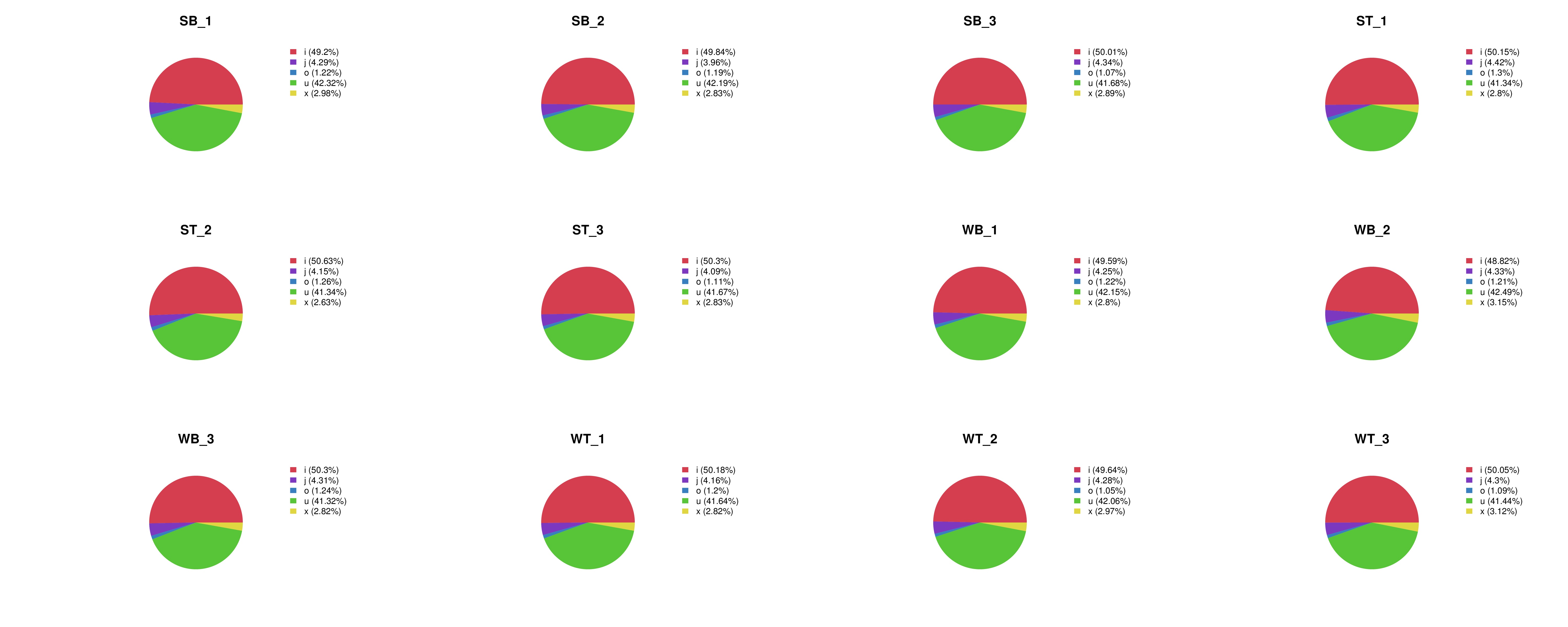

Supplement: Supplementary file 1 [file genes-11-00508-s001.zip › Figure. 1S Pie chart of different class codes of lncRNA in each sample..jpg]
